# In-Vitro Evaluation of Different Antimicrobial Combinations with and without Colistin Against Carbapenem-Resistant *Acinetobacter Baumannii*

**DOI:** 10.3390/molecules24050886

**Published:** 2019-03-03

**Authors:** Alessandra Oliva, Stefania Garzoli, Massimiliano De Angelis, Carolina Marzuillo, Vincenzo Vullo, Claudio M. Mastroianni, Rino Ragno

**Affiliations:** 1 Department of Public Health and Infectious Diseases, Sapienza University, P.le Aldo Moro 5, 00185 Rome, Italy; massimiliano.deangelis@uniroma1.it (M.D.A.) carolina.marzuillo@uniroma1.it (C.M.); vincenzo.vullo@uniroma1.it (V.V.); claudio.mastroianni@uniroma1.it (C.M.M.); 2 Department of Drug Chemistry and Technology, Sapienza University, P.le Aldo Moro 5, 00185 Rome, Italy; stefania.garzoli@uniroma1.it; 3 Rome Center for Molecular Design, Sapienza University, P.le Aldo Moro 5, 00185 Rome, Italy; 4 Alchemical Dynamics s.r.l., 00125 Rome, Italy

**Keywords:** carbapenem-resistant *A. baumannii*, colistin, vancomycin, antimicrobial combinations, synergy, time-kill studies

## Abstract

Carbapenem-resistant *Acinetobacter baumannii* (CR-Ab) infections are associated with high morbidity and mortality. The aim of the study was to evaluate the in-vitro activity of different antimicrobial combinations (with and without colistin, COL) against clinical isolates of CR-Ab collected from patients with CR-Ab infection, including unconventional combinations such as COL + VANcomycin (VAN) and COL + rifampin (RIF). CR-Ab strains were collected from hospitalized patients at Sapienza University of Rome. Antimicrobial susceptibility patterns were determined throughout MIC50/90s whereas the synergistic activity was evaluated by qualitative (i.e., checkerboard) and quantitative (i.e., killing studies) methods. All the strains were found oxacillinase (OXA) producers and tigecycline (TIG) sensitive whereas 2 strains were resistant to COL. Application of the checkerboard method indicated complete synergism in COL combinations at different extension: 21.4%, 57.1%, 42.8%, 35.7% for COL + meropenem (MEM), COL + RIF, COL + VAN and COL + TIG, respectively, with the non-conventional combinations COL + VAN and COL + RIF exhibiting the highest rate of synergism. Regarding COL-free combination, complete synergism was observed in 35.7% of the strains for MEM + TIG. Killing studies showed that the combinations COL + MEM, COL + TIG and MEM + TIG were bactericidal and synergistic against both colistin-sensitive and low colistin-resistant strains whereas only the combinations COL + VAN and COL + RIF showed an early and durable bactericidal activity against all the tested strains, with absence of growth at 24 h. This study demonstrated that COL-based combinations lead to a high level of synergic and bactericidal activity, especially COL + VAN and COL + RIF, even in the presence of high level of COL resistance.

## 1. Introduction

The rapid spread of multidrug-resistant bacteria (MDR) such as carbapenem-resistant (CR) *Enterobacteriaceae (CR-E)*, CR *Acinetobacter baumannii* (CR-Ab) and MDR *Pseudomonas aeruginosa* (MDR-Pa) has become a public health concern, especially in some countries where the diffusion of carbapenem-resistant microorganisms is nowadays endemic [1,2]. In particular, CR-Ab represents a major challenge for physicians due to the high mortality rates especially in the ICU and its capability to persist in the environment [3,4,5].

Therapeutic options are severely limited [2,6] and are mainly based on colistin-containing combinations. However, the ability of CR-Ab to acquire resistance even to colistin (COL) further limits, if not completely precludes, the therapeutic choices. In this scary scenario, the lack of activity of new antimicrobials nowadays available in many countries imposes new efforts in finding the best therapeutic approach against this pathogen [7]. Furthermore, although in the pipeline there are several promising agents with activity towards all CR microorganisms including CR-Ab (i.e., cefiderocol), they are still in in early clinical development of and will be eventually available only in the coming years [8].

Polymyxins at the moment are the antimicrobials with the greatest level of in-vitro activity against CR-Ab [9]. In the studies evaluating the synergistic activity of COL-containing combinations, results suggested that COL plus carbapenems or COL plus antimicrobials active only against Gram-positive bacteria, such as vancomycin (VAN) and rifampin (RIF) can be considered the most active in-vitro combinations [10,11,12].

However, the potential survival benefit obtained with combination therapy instead of monotherapy in infections caused by CR-Ab is still debated, following a recent randomized clinical trial resulted with no advantages of COL combined with meropenem (MEM) versus COL monotherapy for severe CR-Ab infections [13]. On the other hand, the use of COL-based combination (i.e., COL plus RIF) gave a higher rate of microbiological eradication compared with that obtained using monotherapy [14], furthermore, the most likely possibility of developing resistance during COL monotherapy should be taken into account, possibly contributing to the rationale for combination use in CR-Ab infections.

Apart from resistance, which has been increasingly reported in the last years [15], the use of COL in the clinical practice might be limited by its toxic effect; therefore, developing alternative and effective COL-free combinations are of prime importance [16].

Based on the above mentioned studies, the aim of the research reported herein was to evaluate the in-vitro activity of antimicrobial combinations with and without COL against clinical strains of CR-Ab with different clonal types, with particular emphasis on the non-conventional regimens containing COL plus VAN or RIF. In addition, clinical and demographic data of patients were evaluated.

## 2. Results

### 2.1. Bacterial Strains and Clinical Data Collection

Overall, 14 patients with CR-Ab infection (3 bloodstream infections; 2 skin and soft tissue infection; 9 hospital-acquired pneumonia) were included in the study. Clinical characteristics and outcomes of patients are shown in Table 1. All the patients received colistin-based combinations [10: COL + high dosage (i.e., 6 grams/d) of MEM; 2: COL +TIG; 2: COL + RIF], with a total of 6/14 (43%) also receiving VAN. The overall clinical cure was 12/14 (85.7%). CR-Ab strains were isolated from upper/lower respiratory tract fluids (*n* = 9), blood (*n* = 3) and purulent drainages (*n* = 2).

### 2.2. Molecular Typing by Pulsed-Field Gel Electrophoresis

**Figure 1:**
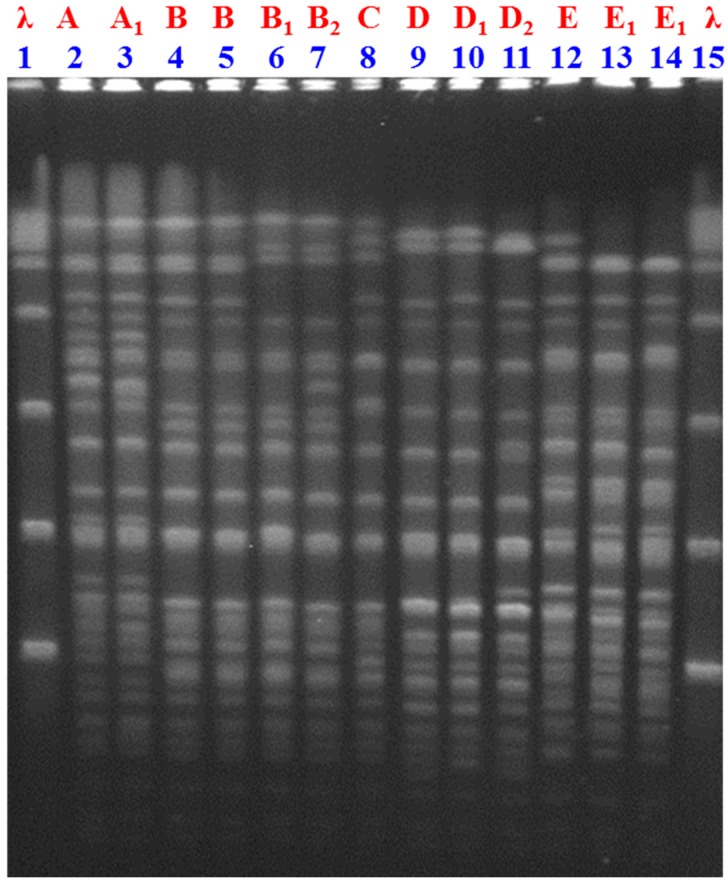
ApaI pulse-field gel electrophoresis (PFGE) patterns of *A baumannii*. Lanes 1, 15 contain molecular size patterns (lambda ladder). Lanes 2,3 (PFGE pattern A), 4–7 (B), 8 (C), 9–11 (D) and 12–14 (E). Isolates with more than 3 fragment variations were assumed to represent strains with major patterns (assignment of capital letters), while isolates with 1–3 fragment differences were considered subtypes (capital letters with numerical subscripts).

Pulsed-field gel electrophoresis (PFGE) analysis was reliable in 13 out of the 14 CR-Ab isolated strains. Overall, 5 clones were detected: A, B, C, D, E (Figure 1). Among these, clone B was the most common (*n* = 4) and was found exclusively in the SUR Paediatric Intensive Care Unit (PICU) whereas clone C was detected in only one strain isolated from a patient hospitalized in a different Intensive Care Unit before being transferred to SUR hospital. Clones A, D and E were found in isolated patients hospitalized in different wards of the hospital (i.e., Internal Medicine, Gerontology and Pneumology).

### 2.3. Antimicrobial Activity

Throughout the VITEK-2 system, all the strains were carbapenem-resistant (MIC > 16mg/L) whereas Table 2 summarizes the in-vitro susceptibility of CR-Ab throughout MBD and E-test methods. Throughout MBD, MICs50/90 were 128/256 mg/L for MEM, 64/128 mg/L for VAN, 256/512 mg/L for GEN, 0.5/1 mg/L for TIG, 0.5/2 mg/L for COL and 16/512 mg/L for RIF. In particular, 2 strains were resistant to COL [1 with low-level of resistance (COL-LR, MIC 4 mg/L), 1 with high-level of resistance (COL-HR, MIC 256 mg/L)]. All the strains were oxacillinase (OXA) producers.

Complete synergism was observed in 3 out of 14 (21.4%), 8 out of 14 (57.1%), 6 out of 14 (42.8%) and 5 out of 14 (35.7%) strains using combinations COL + MEM, COL + RIF, COL + VAN and COL + TIG, respectively. Among COL-free combinations, MEM + TIG showed complete synergism in 5/14 (35.7%) (Table 3).

**Figure 2:**
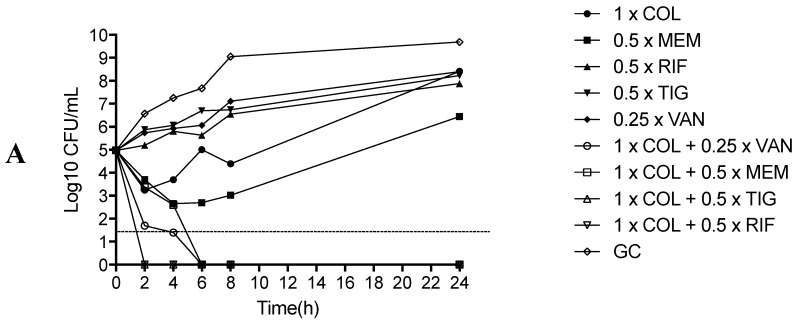

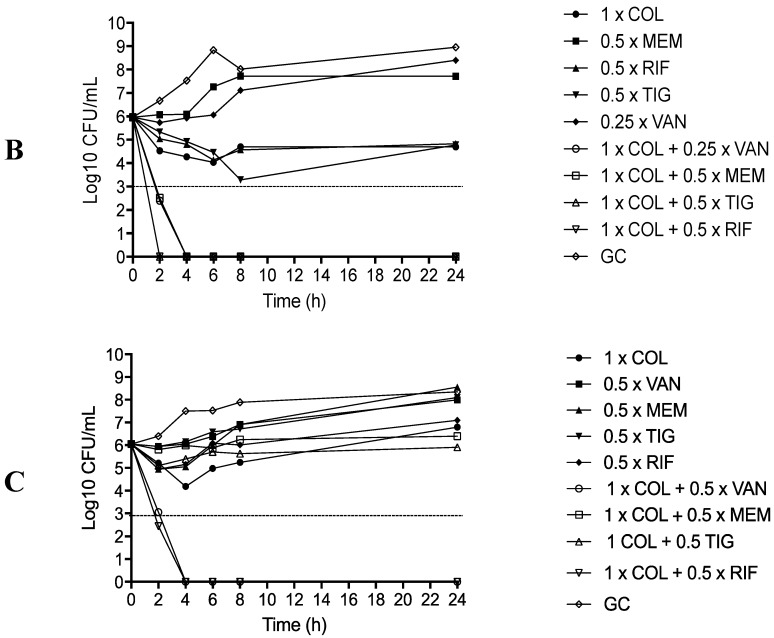
Killing studies of COL-containing combinations against COL-S (1A), COL-LR (1B) and COL-HR (1C) CR-Ab strains. COL: colistin; VAN: vancomycin; MEM: meropenem; TIG: tigecycline; RIF: rifampin; GC: growth control. Dashed line represents bactericidal activity that occurred at any time point compared to the initial bacterial inoculum [17,18,19,20].

**Figure 3:**
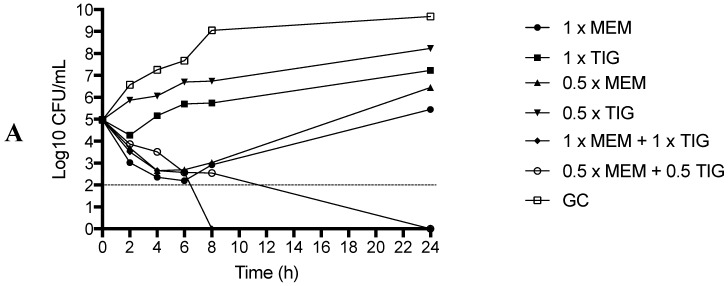

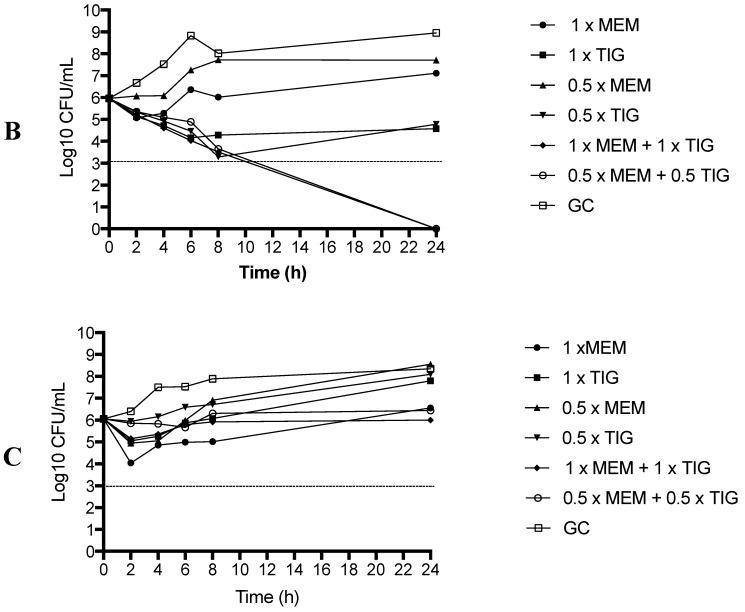
Killing studies of combination without COL against COL-S (2A), COL-LR (2B) and COL-HR (2C) CR-Ab strains. MEM: meropenem; TIG: tigecycline; GC: growth control. Dashed line represents bactericidal activity that occurred at any time point compared to the initial bacterial inoculum.

In killing studies, monotherapy showed a significant growth at 24 h in both colistin-sensitive and colistin-resistant strains. With regard to colistin-based combinations, COL + MEM and COL + TIG were bactericidal and synergistic against both colistin-sensitive and low colistin-resistant strains whereas only the combinations COL + VAN and COL + RIF showed an early and durable bactericidal activity against all the tested strains (including the high colistin-resistant one), with absence of growth at 24h (Figure 2A–C). On the other hand, the combination MEM + TIG showed a bactericidal and synergistic activity at 24 h at the concentrations 1 × MIC MEM + 1 × MIC TIG and 0.5 × MIC MEM + 0.5 × MIC TIG for the colistin-sensitive and the low-level of colistin-resistance strains whereas no effect at all was observed for the high-level of colistin-resistance strain (Figure 3A–C).

Regarding a possible relationship between the clonality of isolates and the synergistic effect of different combinations against CR-Ab, we could say that no synergism was observed for clone A whereas all the tested combinations were synergic towards clone C. MEM + TIG was mostly in-vitro effective against strains belonging to Clone B (3/4, 75%) whereas the un-conventional regimen COL + RIF represented the most synergic combination towards clones D and E (2/3 each, 66%).

## 3. Discussion

Infections caused by CR-Ab represent a major challenge for physicians due to the high morbidity and mortality rates [1]. In particular, the growing levels of resistance even to COL, which has been considered as the last resort drug, is particularly worrying due to the already limited therapeutic options available [21]. In this critical scenario, researchers focused their attention on non-conventional antimicrobial combinations (i.e., COL plus antimicrobials active only against Gram-positives) [11,12] as well as on non-antimicrobial compounds exhibiting antibacterial properties, such as phages or essential oils [22].

With regard to non-conventional antimicrobial combinations, COL + VAN or COL + RIF gained interest given the high activity in-vitro as well as in animal models [23,24] or even in clinical studies [14,25,26,27]. While there is concern regarding the possible augmented risk of nephrotoxicity with the combination COL + VAN [26], the association COL + RIF showed a higher microbiological eradication rate than COL alone, although not influencing the overall mortality compared to COL monotherapy [14]. From a clinical point of view, a recent randomized clinical trial suggested no advantages of COL + MEM combination versus COL monotherapy for severe CR-Ab infections; however, from a microbiological standpoint, the possibility of evinced resistance to COL during monotherapy is a matter of debate and should be taken into account when considering the optimal regimen (monotherapy versus combination therapy) against CR-Ab [13,28].

In the present study, the aim was to investigate on the in-vitro synergistic and bactericidal activity of different antimicrobial combinations (with and without COL) against strains of CR-Ab isolated from patients with CR-Ab infections whose clinical data were also presented. Herein, it has been demonstrated that a combination consisting of COL + VAN or COL + RIF was effective against CR-Ab and might represent a valid therapeutic option for severe infections caused by MDR *A. baumannii.*

Interestingly, the results of killing studies showed that these combinations were highly effective even in the presence of COL resistance. This finding is in line with previous in-vitro studies that evaluated different combinations against COL-resistant strains and found that COL associated with RIF was the most synergistic one [29,30,31,32,33,34]. However, the strength of the present study resides on the evaluation of the synergistic effect of the tested combinations, including COL + VAN, by killing studies rather than other methods, thus giving additional information on the kinetic for obtaining the bactericidal and synergistic activity. In fact, it is herein shown that the bactericidal effect of COL + RIF and COL + VAN occurred early, after 2–4 h of incubation, in the absence of bacterial growth for up to 24 h. The rationale of using COL + VAN or RIF relies on COL cell-permeabilizing properties, which might allow associated drug to reach its target at sub-inhibitory concentrations [10,35,36].

Apart from in-vitro results, the clinical experience on the use of these regimens against COL-resistant CR-Ab is still low [15,37]. Thus, in addition to microbiological analyses, in the present report clinical data of patients infected with CR-Ab are also presented, including subjects with infections due to COL-resistant strains who received treatment based on COL + VAN or COL + RIF combinations. Herein, the overall mortality was low and did not differ between patients treated with COL + VAN, COL + RIF or other COL-based combinations, with absence of nephrotoxicity in patients receiving COL + VAN.

A further strength of the study is the investigation of combinations not containing COL, such as MEM + TIG; in fact, this combination could still retain a place in therapy when the use of COL is contraindicated or the risk of COL pharmacokinetic under exposure is a matter of concern [16]. Overall, we found a rate of synergy slightly higher than that found in previous studies by the checkerboard method (36% vs. 20%), which was most prominent in COL sensitive strains [15,38]. In the killing studies, although MEM + TIG was in-vitro effective against COL-sensitive strain, in the presence of low-level of COL resistance the bactericidal activity, although still present, occurred only after 24 h and, in the presence of high-level of COL resistance, the activity was completely absent. The latter is of particular importance due to main points: i) in the presence of kidney failure or other side effects related to the use of COL, MEM + TIG combination might be considered a better option to treat COL susceptible CR-Ab strains whereas in the presence of high COL resistance this combination may not be effective; ii). The presence of COL resistance represents a condition which might make the isolates more susceptible to COL-containing regimens, probably as a consequence of the alterations in the outer membrane and lipopolysaccharide (LPS) that confer COL resistance, thus raising the vulnerability of CR-Ab to these combinations [39]. However, a definite conclusion on the therapeutic role of MEM + TIG combination in the presence of COL-resistance cannot be deduced with certainty due to the low number of COL-resistant strains tested.

With regard to the results of PFGE analyses, it was shown that clone B was the most common and exclusively found in one specific ward, whereas clone C belonged to a strain not primarily circulating at SUR hospital. On the other hand, the diffusion of clones A, D and E in wards not related to each other suggests that multiple clones of CR-Ab are simultaneously present in the hospital. In an attempt to evaluate whether the synergistic effect against CR-Ab was related to the clonality of the isolates, we found that all the tested combinations did not show the same synergistic effect against different clones of CR-Ab, with some apparently more effective than others towards a specific clone. However, given the paucity of tested strains, a definite conclusion on the potential role of clonality on determining the synergistic effect of different combinations cannot be drawn [15].

## 4. Materials and Methods

### 4.1. Bacterial Strains and Clinical Data Collection

A series of CR-Ab collected from patients hospitalized at Sapienza University of Rome (SUR) was included in the study. Clinical samples underwent traditional microbiological procedures as for daily practice, with the use of the VITEK-2 (Bio-Merieux, Marcy l’Etoile, France) automated system for identification and antimicrobial susceptibility testing. Until further microbiological analyses, bacteria were stored on cryovial bead preservation system (Microbank; Pro-Lab Diagnostics, Richmond Hill, Ontario, Canada) at −80 °C. Overnight cultures were then adjusted to a turbidity of 0.5 McFarland, corresponding to ≈1 × 10^8^ CFU/mL. For each patient clinical and demographic data were collected. Type of infection was defined in accordance to Centres for Disease Control and Prevention (CDC) guidelines [40]. Clinical cure was defined as resolution of CR-Ab infection. Data collection and microbiological analysis were anonymously and confidentially performed and approved by the Ethics Committee of Policlinico Umberto I of Rome. All experiments were performed in accordance with guidelines and regulations, following the rules of the Declaration of Helsinki.

### 4.2. Antimicrobial Agents

Antimicrobial agents were provided as purified powders by the manufacturer (Sigma Aldrich, Milan, Italy). Stock solutions at different concentrations were prepared in sterile and pyrogen-free 0.9% saline or water, according to manufacturer’s instructions.

### 4.3. Molecular Typing by Pulsed-Field Gel Electrophoresis (PFGE)

The preparation of genomic DNA of CR-Ab isolates was performed as previously described [41]. DNA restriction was done with ApaI enzyme (New England Biolabs, Ipswich, MA, USA) at 25 °C for 4 h. The gels were run on a CHEF-DRII system (Bio-Rad Laboratories, Hercules, CA, USA) over 20 h at 14 °C with 5 to 13 s of linear ramping at 200 V. It was assumed that a single base mutation in the chromosomal DNA could introduce at most a 3-fragment difference in the restriction pattern [42]; isolates with more than 3 fragment variations were assumed to represent strains with major patterns (assignment of capital letters), while isolates with 1–3 fragment differences were considered subtypes (capital letters with numerical subscripts).

### 4.4. Antimicrobial Evaluation

Minimal inhibitory concentrations (MICs) of MEM, COL, RIF, tigecycline (TIG) and VAN were determined by macro broth dilution (MBD) method in cation-adjusted Mueller Hinton broth (CA-MHB) [43]. Briefly, two-fold serial dilutions of each antimicrobial agent were prepared in 2 mL CA-MHB in borosilicate glass tubes and incubated for 24 h at 37 °C. MIC was defined as the lowest concentration of antibiotic that completely inhibited visible growth. In addition, for COL and TIG a gradient strip MIC determination (E-test) was performed. For synergistic activity evaluation, checkerboard method of the following antimicrobial combinations was performed: COL + MEM, COL + RIF, COL + TIG, COL + VAN, MEM + TIG. A 96-well microtiter plate containing antibiotic combinations at different concentrations was used to perform checkerboard synergy testing. Wells containing a final inoculum of ~5 × 10^5^ CFU/mL were incubated at 37 °C for 24 h under static conditions in CA-MHB. The fractional inhibitory concentration index (FICI) of each combination was defined as: ∑FIC: FICA + FICB = MICA + B/MICAalone + MICB + A/MICBalone. Synergism was defined as FICI ≤ 0.5 whereas FICI > 0.5 but <4 were considered as indifferent or non-antagonistic [44]. In addition, killing studies of different antimicrobial combinations were performed on 3 representative strains, according to COL sensitivity [one strain with full COL susceptibility (COL-S); one strain with low-level of COL resistance (COL- LR); one strain with high-level of COL resistance (COL-HR)]. COL susceptibility or resistance was defined in accordance to the European Committee on Antimicrobial Susceptibility Testing (EUCAST) clinical breakpoint [18,19]. The following concentrations were used for killing tests: 1 × MIC COL, 0.5 × MIC RIF, 0.5 × MIC MEM, 0.5 × MIC TIG, VAN, 1 × MIC COL + VAN, 1 × MIC COL + 0.5 × MIC TIG, 1 × MIC COL + 0.5 × MIC RIF, 1 × MIC COL + 0.5 × MIC MEM, 0.5 × MIC MEM + 0.5 × MIC TIG, 1 × MIC MEM + 1 × MIC TIG. The tested VAN concentration was fixed at 16 mg/L, which reflects the serum achievable concentration during VAN therapy [25]. Bactericidal activity was defined as ≥99.9% (i.e., ≥ 3-log10 CFU/mL) reduction of the initial bacterial count at each time point. Synergy was defined as a ≥100-fold decrease in CFU/mL between the combination and its most active constituent at the same concentration after 24 h, with the number of surviving organisms in the presence of the combination ≥ 100-fold CFU/mL below the starting inoculum. The MIC values used for synergy testing were those obtained throughout MBD. All in-vitro experiments were performed in triplicate.

## 5. Conclusions

In conclusion, it is herein demonstrated that combinations of COL with VAN or RIF were highly synergic and rapidly bactericidal against CR-Ab, including COL-resistant strains and that, in the presence of kidney failure or other side effects related to the use of COL, MEM + TIG might be considered a reasonable approach to treat mainly COL susceptible CR-Ab strains.

## Figures and Tables

**Table 1:** Clinical characteristics of patients (*n* = 14) with CR-Ab infection.

Patient	Type of Infection	Therapy	Duration of Therapy (days)	Outcome
1	BSI	C + M + V	14	Cured
2	HAP	C + M + V	13	Cured
3	SSI	C + T + V	30	Cured
4	SSI	C + R	14	Cured
5	BSI	C + M	30	Cured
6	HAP	C + V + R	14	Cured
7	BSI	C + M + V	6	Died*
8	HAP	C + M	17	Cured
9	HAP	C + M	14	Cured
10	HAP	C + M	14	Cured
11	HAP	C + M	14	Cured
12	HAP	C + T	14	Cured
13	HAP	C + M	10	Died*
14	HAP	C + M + V	14	Cured

BSI: Bloodstream infection. SSI: Skin and Soft Tissue Infection. HAP: Hospital-acquired pneumonia. *: pt#7 died because of CR-Ab infection; pt#13 died for reason other than CR-Ab infection. C: colistin; M: meropenem; V: vancomycin; R: rifampin; T: tigecycline.

**Table 2:** In-vitro susceptibility of carbapenem-resistant *Acinetobacter baumannii* (CR-Ab) throughout macrobroth dilution (MBD) and E-test methods.

	Antibiotic	MEM	COL		VAN	RIF	GEN	TIG	
	Method	MBD	MBD	E-Test	MBD	MBD	MBD	E-Test	MBD
Patients/Clinical Isolates	1	128	1.000	0.750	256	8	4	2.00	0.50
	2	256	0.250	0.300	32	4	>512	1.50	1.00
	3	256	0.060	0.380	64	>512	2	0.75	1.00
	4	128	0.032	0.075	128	>512	>512	2.00	0.50
	5	32	0.125	0.380	64	4	>512	1.00	1.00
	6	128	4.000	4.00	64	4	512	3.00	0.50
	7	512	0.015	0.750	128	>512	>512	2.00	1.00
	8	256	0.030	0.380	128	4	32	1.50	0.25
	9	256	0.250	0.500	128	4	>512	2.00	0.50
	10	32	2.000	1.000	128	2	>512	1.50	0.50
	11	128	0.500	1.500	64	16	64	0.75	0.75
	12	8	256.0	128.0	32	>512	2	1.00	0.25
	13	64	0.125	0.500	32	4	>512	1.50	0.25
	14	32	0.500	0.500	64	4	256	2.00	0.50

MEM: meropenem; COL: colistin; VAN: vancomycin; RIF: rifampin; GEN: gentamicin; TIG: tigecycline.

**Table 3:** Synergism of different combinations against CR-Ab throughout checkerboard method. The 14 clinical isolated as listed in Table 1 were used.

Strains	PFGE Pattern	COL + MEM		COL + RIF		COL + VAN		COL + TIG		MEM + TIG	
		FICI°	Syn	FICI	Syn	FICI	Syn	FICI	Syn	FICI	Syn
1	B	0.375	S	0.375	S	0.250	S	0.375	S	1.000	I
2	B_1_	0.625	I	0.375	S	0.625	I	1.000	I	0.500	S
3	B	0.625	I	0.625	I	0.750	I	0.625	I	0.375	S
4	A	0.750	I	0.625	I	0.625	I	1.000	I	0.750	I
5	D	0.625	I	0.500	S	0.250	S	0.250	S	0.625	I
6 *	C	0.250	S	0.250	S	0.250 °	S	0.250	S	0.375	S
7	A_1_	1.000	I	1.000	I	1.000	I	2.000	I	0.625	I
8	E_1_	0.625	I	0.750	I	0.375	S	2.000	I	1.000	I
9	B_2_	0.750	I	0.750	I	0.625	I	2.000	I	0.250	S
10	E	0.250	S	0.250	S	0.250	S	0.250	S	0.625	I
11	-	1.000	I	0.250	S	0.375	S	0.375	S	0.375	S
12 **	D_2_	0.750	I	0.250	S	0.625 ^§^	I	2.000	I	1.000	I
13	D_1_	2.000	I	0.625	I	0.625	I	2.000	I	0.625	I
14	E_1_	0.625	I	0.250	S	0.625	I	0.750	I	0.625	I
Total n			3/14		8/14		6/14		5/14		5/14
Total (%)			21.4		57.1		42.8		35.7		35.7

*: strain with low-level of COL-resistance (COL-LR, MIC 4 mg/L); **: strain with high-level of COL-resistance (COL-HR, MIC 256 mg/L); °: FICI 0.250 was obtained at the following concentrations: 0.5 mcg/mL COL (0.125 × MIC)+ 8 mcg/mL VAN (0.125 × MIC); ^§^: FICI 0.625 was obtained at the following concentrations: 32 mcg/mL COL (0.125 × MIC) + 16 mcg/mL VAN (0.5 × MIC). The fractional inhibitory concentration index (FICI) of each combination was defined as: ∑FIC: FICA + FICB = MICA + B/MICAalone + MICB + A/MICBalone. Synergism was defined as FICI ≤ 0.5 (S) whereas FICI > 0.5 but <4 were considered as indifferent (I). COL: colistin; MEM: meropenem; RIF: rifampin; VAN: vancomycin; TIG: tigecycline.
